# Electroless etching of Si with IO_3_^–^ and related species

**DOI:** 10.1186/1556-276X-7-323

**Published:** 2012-06-20

**Authors:** Kurt W Kolasinski, Jacob W Gogola

**Affiliations:** 1 Department of Chemistry, West Chester University, West Chester, PA, 19383, USA; 2 Present address: Chemistry Department, Exelon Corporation, Plant Services Building, 1848 Lay Road, Delta, PA, 17314, USA

**Keywords:** Porous silicon, Etching, Nanostructures, Thin films, Hydrofluoric acid, Iodate, Iodine, Iodide, Triiodide, 81.07.-b, 81.65.Cf, 68.55.ag

## Abstract

We have previously derived seven requirements for the formulation of effective stain etchants and have demonstrated that Fe^3+^, Ce^4+^, and VO_2_^+^ + HF solutions are highly effective at producing nanocrystalline porous silicon. Here, we show that Cl_2_, Br_2_, I_2_, ClO_3_^–^, BrO_3_^–^, IO_3_^–^, I^–^, and I_3_^–^ induce etching of silicon when added to HF. However, using the strict definition that a pore is deeper than it is wide, we observe little evidence for porous layers of significant thickness but facile formation of pits. Iodate solutions are extremely reactive, and by the combined effects of IO_3_^–^, I_3_^–^, I_2_, and I^–^, these etchants roughen and restructure the substrate to form a variety of structures including (circular, rectangular, or triangular) pits, pyramids, or combinations of pits and pyramids without leaving a porous silicon layer of significant thickness.

## Background

Stain etching is potentially a fast, easy, and inexpensive method of producing porous silicon (por-Si) on arbitrarily shaped surfaces. When mixed with HF(aq), Fe^3+^, Ce^4+^, and VO_2_^+^ have all previously been shown [1-5] to be capable of producing uniform layers of por-Si with thicknesses up to 20 μm. Perhaps more importantly for applications, the ability to etch arbitrarily shaped substrates means that stain etchants can also efficiently etch powdered silicon. Loni et al. [6] have demonstrated that inexpensive metallurgical-grade silicon powder can be converted to high-surface-area por-Si powder. Porous silicon powder may find applications as a high-energy material [7], in drug delivery [8], biosensing [9], and as an anode material in lithium-ion batteries [10].

When silicon is exposed to an aqueous fluoride solution, the morphology of the etched surface depends sensitively on the balance of the surface reactions that occur. Oxidation by water dissociation or step flow etching initiated by OH^–^ both favor the formation of uniform surfaces, whereas direct Si atom dissolution via the Gerischer mechanism [11,12] favors por-Si formation. Surface chemistry alone does not lead to por-Si formation. The chemistry must be coupled to charge carrier dynamics to form a porous film. In electroless etching to form por-Si (stain etching), the quantum confinement-related shift of the Si valence band causes fluoride etching of Si to be self-limiting, which is responsible for nanostructure formation [13].

We have undertaken a series of experiments to develop new stain etchants and better understand the dynamics of etching [1-5]. The seven requirements for the formulation of an effective stain etchant are [13,14] (1) an acidic fluoride solution, (2) sufficiently high fluoride concentration compared to the oxidant concentration, (3) the oxidant must be able to inject holes into the Si valence band at an appreciable rate; thus, its electrode potential should be more positive than approximately +0.7 V, (4) oxide formation needs to be slow or nonexistent, (5) the oxidant and all products are soluble, (6) film homogeneity is enhanced if the oxidant's half-reaction does not evolve gas, and finally, (7) the net etching reaction from hole injection to Si atom removal (including the reactions of any by-products) has to be sufficiently anisotropic (attacking all kinds of sites but only at the bottom of the pore) to support pore nucleation and propagation.

Previous work has shown that etching of silicon is affected by the addition of halogens and halogenates to fluoride solutions. Salem et al. [15] were able to increase the rate of anodic groove formation caused by etching at the contact of a Pt wire with a Si substrate by adding 1 mM I_2_ to the etchant. Seo and co-workers investigated etching induced by KIO_3_ and KBrO_3_[16-18]. However, alkali metals precipitate readily with the etch product SiF_6_^2–^[19]. Precipitation hinders the reproducible formation of uniform layers, and no significant por-Si layers were observed in these studies.

Adachi and co-workers have performed a number of studies with iodates and other oxidants. Xu and Adachi [20] investigated etching KIO_3_ + HF solutions with and without illumination at ≥600 nm from a Xe lamp. They found no formation of por-Si in the absence of illumination. When illuminated, they attributed observed photoluminescence (PL) to microporous layers with a thickness of ≤80 nm. However, it should be noted that PL has previously been observed from the interface of hexafluorosilicates with silicon [19] and that the lateral size (20 to 100 nm) of features they observed by atomic force microscopy was often larger than the reported film thickness. They also report that por-Si emitting stable red PL can be formed anodically in the presence of the very low concentrations of I_2_ provided by a saturated I_2_/50% HF solution [21]. During anodic etching of n-type wafers [22], the addition of KIO_3_ increased the observed pore size. Their report that 10^–4^ M KIO_3_ solutions do not react with Si surfaces runs counter to the high reactivity reported here for HIO_3_ solutions.

Halogenate + HF solutions can be made that meet all of the criteria for stain etchants except the last. Therefore, these species are not suitable for por-Si formation even though they very effectively induce etching and restructuring of the surface. On the other hand, we demonstrate that HIO_3_ + HF is quite capable of restructuring the surface to produce other sorts of nano- and micro-structures.

## Methods

Diffuse reflectance infrared spectroscopy, cross-sectional scanning electron microscopy, photoluminescence, and white light reflectometry have been used to measure the thickness, surface area, and porosity of por-Si thin films and restructured Si substrates made by oxidant + HF etching in various solutions as a function of solution composition and etch time. Scanning electron microscopy (SEM) was performed with an FEI Quanta 400 ESEM (Hillsboro, OR, USA). The SEM operates with integrated Oxford INCA energy-dispersive X-ray spectroscopy (Abingdon, UK). Fourier transform infrared spectroscopy was performed as described previously [2] with a Nicolet Protégé 460 (Madison, WI, USA). Reflectometry was performed on an Ocean Optics USB2000 spectrometer with a DH-2000 deuterium tungsten halogen lamp from 360 to 1,100 nm (Dunedin, FL, USA). Photoluminescence measurements were made on a Cary Eclipse fluorescence spectrophotometer with excitation at 350 nm (Agilent Technologies, Inc., Santa Clara, CA, USA).

All etching was performed on either 0- to 100-Ω cm or 14- to 20-Ω cm p-type test-grade Si(100) wafers, or mechanical-grade n-type Si(111) wafers (University Wafer, Boston, MA, USA). The wafers are cleaned by ultrasonication in acetone then ethanol followed by rinsing in water prior to etching. Exposure of the samples to air after cleaning is minimized. After etching, samples are rinsed in water and ethanol, and then dried in a stream of Ar gas.

Table 1 lists the standard electrochemical potentials of a number of oxidants of interest. We have shown previously [1-5] that Fe^3+^, Ce^4+^, and VO_2_^+^ are quite capable for facile production por-Si but HCrO_4_^–^ and MnO_4_^–^ are less reliable for por-Si formation. Both are capable of inducing etching. Chromate is particularly effective at etching defects in silicon [23]. Thus, its solutions may exhibit too much anisotropy for uniform por-Si formation. Permanganate is usually supplied from a Na or K salt, and as mentioned above [19], alkali metals precipitate readily with the etch product SiF_6_^2–^, which can lead to interferences and inhomogeneities.

**Table 1:** **Standard *E° *of oxidants of interest and approximate positions of VBM at the silicon surface**

**Species**	**E°/V**	**Half-reaction**
${\text{I}}_{2}$	0.5355	${\text{I}}_{2}+2{\text{e}}^{-}\to {2\text{I}}^{-}$
VBM (n, surface)	0.67	
${\text{Fe}}^{3+}$	0.771	${\text{Fe}}^{3+}+{\text{e}}^{-}\to {\text{Fe}}^{2}$
${\text{IrC}1}_{6}^{2-}$	0.8665	${\text{IrC}1}_{6}^{2-}+{\text{e}}^{-}\to {\text{IrC}1}_{6}^{3-}$
VBM (p, surface)	0.9	
${\text{NO}}_{3}^{-}$	0.957	${\text{NO}}_{3}^{-}+4{\text{H}}^{\text{+}}\;\text{+}\;{3\text{e}}^{-}\to \text{NO(g})\;\text{+}\;{\text{H}}_{2}\text{O}$
${\text{VO}}_{2}^{+}$	0.991	${\text{VO}}_{2}^{+}+2{\text{H}}^{+}+{\text{e}}^{-}\to {\text{VO}}^{2+}+{\text{H}}_{2}\text{O}$
${\text{Br}}_{2}$	1.0873	${\text{Br}}_{2}+2{\text{e}}^{-}\to 2{\text{Br}}^{-}$
${\text{IO}}_{3}^{-}$	1.195	${\text{IO}}_{3}^{-}+6{\text{H}}^{+}+5{\text{e}}^{-}\to \frac{1}{2}\;{\text{I}}_{2}+3{\text{H}}_{2}\text{O}$
${\text{HCrO}}_{4}^{-}$	1.350	${\text{HCrO}}_{4}^{-}+7{\text{H}}^{+}+3{\text{e}}^{-}\to {\text{Cr}}^{3+}+4{\text{H}}_{2}\text{O}\;$
${\text{Cl}}_{2}$	1.35827	${\text{C}1}_{2\;}+2{\text{e}}^{-}\to 2{\text{C}1}^{-}$
${\text{ClO}}_{3}^{-}$	1.47	${\text{ClO}}_{3}^{-}+6{\text{H}}^{+}+5{\text{e}}^{-}\to \frac{1}{2}\;{\text{C}1}_{2}+3{\text{H}}_{2}\text{O}$
${\text{BrO}}_{3}^{-}$	1.482	${\text{BrO}}_{3}^{-}+6{\text{H}}^{+}+5{\text{e}}^{-}\to \frac{1}{2}{\text{Br}}_{2}+3{\text{H}}_{2}\text{O}$
${\text{MnO}}_{4}^{-}$	1.507	${\text{MnO}}_{4}^{-}+8{\text{H}}^{+}+5{\text{e}}^{-}\to {\text{Mn}}^{2+}+4{\text{H}}_{2}\text{O}$
${\text{Ce}}^{4+}$	1.72	${\text{Ce}}^{4+}+{\text{e}}^{-}\to {\text{Ce}}^{3+}$
${\text{S}}_{2}{\text{O}}_{8}^{2-}$	2.0	${\text{S}}_{2}{\text{O}}_{8}^{2-}+2{\text{e}}^{-}\to 2{\text{SO}}_{4}^{2-}$

*E°*, electrode potentials; VBM, valence band maximum.

Two other oxidants that appear in Table 1 are ${\text{IrCl}}_{6}^{2-}$ and ${\text{S}}_{2}{\text{O}}_{8}^{2-}$. We have found that ${\text{IrCl}}_{6}^{2-}$ is capable of forming por-Si films and will report on this in detail elsewhere. We have attempted a few cursory etching experiments with K_2_S_2_O_8_ or (NH_4_)_2_S_2_O_8_ in 48 wt.% HF. There are signs of reaction, such as bubbling and waviness on the surface during etching, but we have not observed por-Si film formation. Grains of the solid dropped into HF induced vigorous bubbling, which slows markedly once they have dissolved. We are in the process of trying to synthesize H_2_S_2_O_8_. This is a better candidate, as it will not suffer from precipitation interferences.

Here, we concentrate on solutions made up from NaI (Fisher certified; Pittsburgh, PA, USA), NaIO_3_ (Fischer certified), HIO_3_ (Sigma-Aldrich ACS reagent, >99.5%; St. Louis, MO, USA), I_2_ (Fisher USP resublimed), HI (Sigma-Aldrich, 57 wt.% in water, unstabilized), NaBrO_3_ (Fisher certified), Br_2_, HBr, NaClO_3_ (Fisher certified), Cl_2_, and HCl. These are added to 48 wt.% HF (Acros Organics ACS reagent, Fair Lawn, NJ, USA).

## Results and discussion

The iodate ion IO_3_^–^ very effectively injects charge into Si through the five-electron process described in Table 1. The induced etching is highly exothermic and rapid. HIO_3_ concentrations in the range of 5 × 10^–4^ to 1 × 10^–2^ M have all been found to produce rapid etching.

Etching in fluoride solutions leads to the formation of the hexafluorosilicate ion SiF_6_^2–^ and H_2_ bubbles by the reaction scheme [1]

(1) $$\;\{\begin{array}{c} \text{ Si}+{\text{Ox}}^{\text{+}}\to {\text{Si}}^{+}+\text{Ox}\\ {\text{ Si}}^{+}+3\text{HF}\to {\text{HSiF}}_{3}+2{\text{H}}^{+}+{\text{e}}^{-}\\ \frac{{\text{HSiF}}_{3}+3\text{HF}\to {\text{H}}_{2}{\text{SiF}}_{6}+{\text{H}}_{2}}{\text{Si(s})+{\text{Ox}}^{\text{+}}+6\text{HF}\to {\text{H}}_{2}{\text{SiF}}_{6}+\text{Ox}+2{\text{H}}^{+}+{\text{H}}_{2}(\text{g})+{\text{e}}^{-}}\\ {\text{ H}}^{+}+{\text{e}}^{-}\to \frac{1}{2}{\text{H}}_{2}(\text{g}) \end{array}\text{.}$$

For brevity, (aq) is dropped. Thus, as mentioned above, salts containing Na or K should be avoided as a source of IO_3_^–^ to avoid precipitation. Using NaIO_3_, we could easily induce precipitation of Na_2_SiF_6_ with sufficiently extensive etching. Alkali metal hexafluorosilicates are white powders.

**Figure 1:**
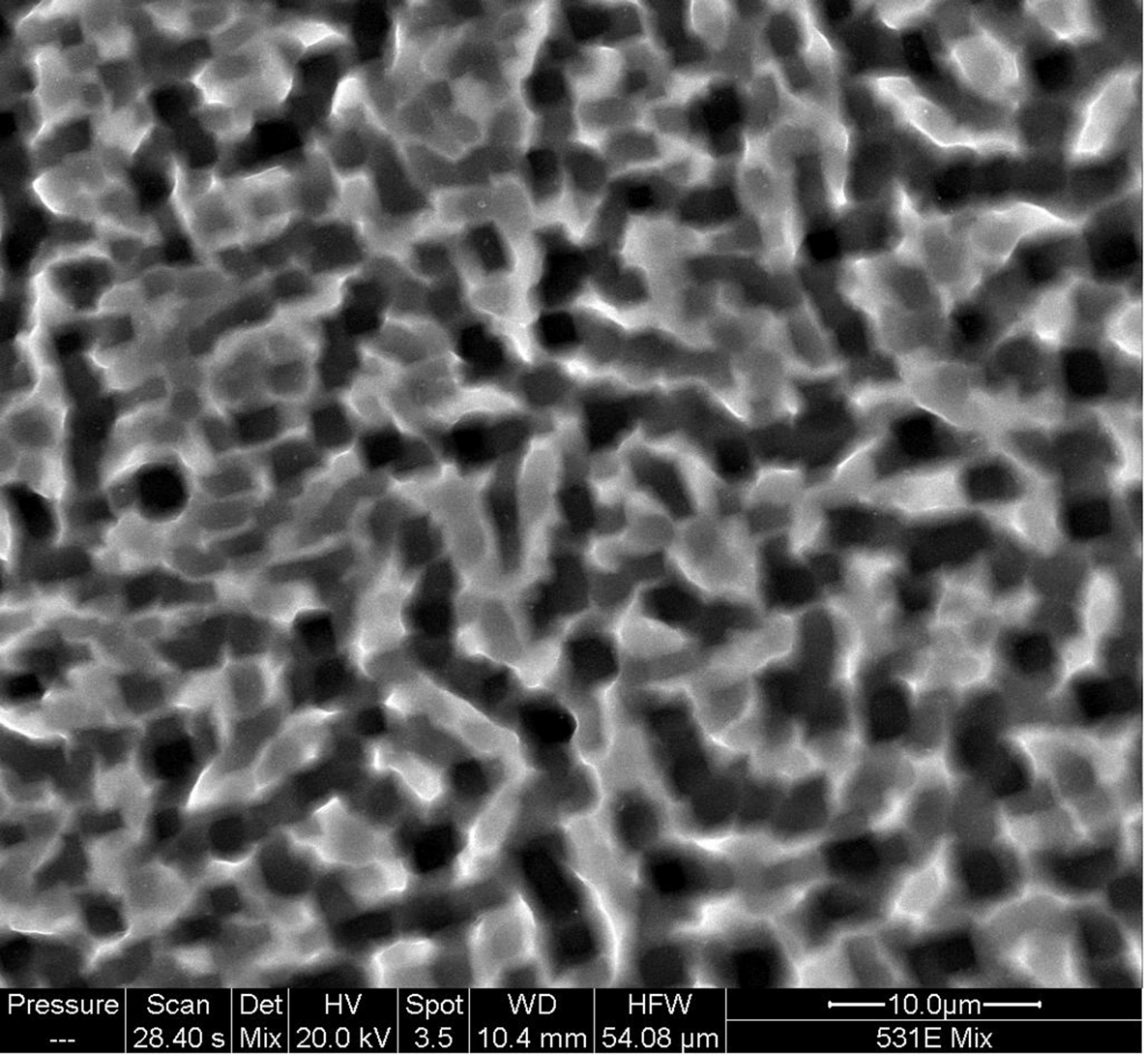
**SEM micrograph of Si(100) substrate etched in 0.002 M HIO**_**3 **_**in 48% HF for 30 min.**

Observing the etching of Si in HIO_3_ + HF, one sees bubble formation on the crystal accompanied by the formation of a rough-looking surface and dark particulates. Rinsing the crystal in ethanol releases the particulates and causes the solution to take on the typical red coloration of a tincture of iodine. Clearly, I_2_ is being formed during etching. Figure 1 displays a typical SEM micrograph of a Si substrate etched in an aqueous solution of HIO_3_/HF. The surface is rough and pitted with no indication of the formation of a nanoporous layer.

The precipitation of I_2_ can be avoided by the addition of ethanol to the etchant. Ethanol addition significantly reduced the etch rate of most stain etchants [2]; however, that was not a problem for the highly reactive iodate system. Bubble formation and roughening were again observed. In this case, the etchant gradually turned red, reached a maximum intensity, and then became clear. At this point, the bubbling also ceased. I_2_ was being produced by the reduction of IO_3_^–^; however, it was also being consumed by a secondary reaction. The dissolution and etch rates can be enhanced by performing the etching with simultaneous ultrasonic agitation.

**Figure 2:**
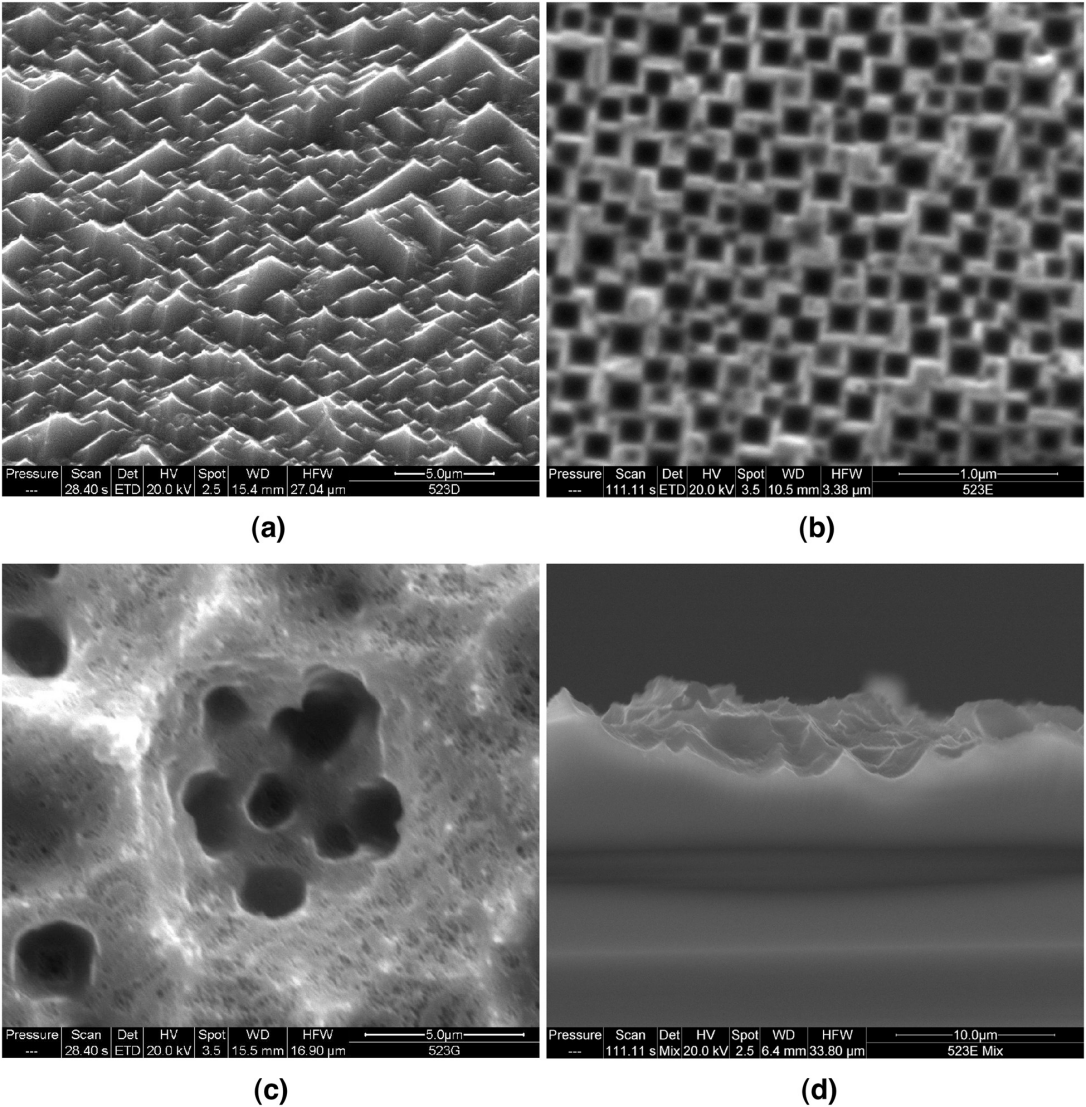
**SEM micrographs of Si(100) wafers etched in 0.07 M HIO**_**3 **_**in 3:5 ethanol/HF solutions.** (**a**) Etch time = 300 s, 45° perspective. (**b**) Etch time = 900 s, plan view. (**c**) Etch time = 1800 s, plan view. (**d**) Cross section of sample shown in (b).

As shown in Figure 2, significant surface restructuring is possible with HIO_3_ + ethanol + HF etching. The surfaces are extremely rough and have a gray to black appearance. The lack of visible photoluminescence and the absence of a significant Si-H peak in the infrared absorption spectrum indicate the absence of a nanoporous layer. The surface features are largely uncontrollable. The same sample can exhibit regions covered with pyramids, as shown in Figure 2a when the substrate is (100) oriented. These pyramids have smooth faces, but in between, there is significant roughness and a significant number of circular pits 45 to 110 nm in diameter. Other regions will exhibit rectangular features as shown in Figure 2b. As the micrograph in panel (d) shows, these roughly 150-nm features are pits rather than pores. If the substrate orientation is switched to (111), triangular rather than rectangular pits are found. This is to be expected for anisotropic etching with etch rates that are dependent on surface orientation as found in the alkaline etching of Si [24]. Other regions exhibit corral-like structures (on both (111) and (100) orientations) with nested pits within pits and significant roughness, as shown in panel (c). Regions such as those shown in panels (b) and (c) or the between-pyramid regions might be labeled por-Si; however, because they do not exhibit a depth significantly longer than their width, large surface area (i.e., the lack of significant Si-H IR absorption), nor photoluminescence, we refrain from designating them as por-Si.

We believe that IO_3_^–^-induced etching is capable of producing nanoporous silicon. This generates a great deal of surface area, which enhances the reactivity of the Si substrate toward other species. This facilitates such major restructuring of the surface because the chemistry of iodine-containing species in solution is extremely complex, and it is impossible to separate IO_3_^–^-induced chemistry from that induced by I^–^, I_2_, and I_3_^–^. As we will show below, all of these species are capable of etching Si. The first two lead to smooth isotropic etching, and the last leads to anisotropic etching. The combination and balance of these chemistries with IO_3_^–^-induced etching leads to the great variety of surface features observed.

There are two possible production paths for I_2_. The first is the reduction of IO_3_^–^ listed in Table 1. The second is reaction (2)

(2) $${\text{IO}}_{{}_{3}}^{-}+5{\text{I}}^{-}+6{\text{H}}^{+}\to 3{\text{I}}_{2}+3{\text{H}}_{2}\text{O}\;E{}^{\circ}=0.6595\;\text{V,}$$

which occurs spontaneously when IO_3_^–^ and I^–^ are mixed. A relatively small amount of I_2_ can be lost to adsorption on the surface [25]. Two possible sinks for I_2_, apart from simple precipitation of I_2_(s), are a thermally initiated etch reaction involving I_2_ as well as a fluoride species (because I_2_ in solution by itself does not etch silicon in solution at room temperature, see below),

(3) $$\text{Si}+x{\text{I}}_{2}^{}+y\text{HF}\to {\text{H}}_{2}{\text{SiF}}_{y}{\text{I}}_{2x}+\frac{1}{2}\left(y-2\right){\text{H}}_{2}(\text{g})\text{,}$$

(with *x* = 1 or 2 and *y* = 4 or 2) and the reaction with iodide to form triiodide,

(4) $${\text{I}}_{2}^{}+{\text{I}}^{-}\to {\text{I}}_{3}^{-}\text{.}$$

The stoichiometry of reaction (3) is notional. By a thermal reaction, we intend to imply that it is initiated by the chemical reaction of I_2_ dissociative adsorption on the surface of silicon as opposed to a hole injection step. Subsequent steps involve some unidentified combination of fluoride species. Because the first step does not involve hole injection, this reaction would not be constrained by quantum confinement effects to be self-limiting [13] and would, therefore, be capable of destroying por-Si. It should be noted that all silicon tetrahalides are unstable in water and subject to reactions of the type

(5) $${\text{SiX}}_{4}^{}+2\text{HF}\to {\text{H}}_{2}{\text{SiX}}_{4}{\text{F}}_{2}\text{.}$$

Iodide production can occur from the reduction of I_2_ listed in Table 1 (though the rate of this should be low based on the *E°* value), the reduction of triiodide,

(6) $${\text{I}}_{3}^{-}+2{\text{e}}^{-}\to 3{\text{I}}^{-}\;E{}^{\circ}=0.536\;\text{V,}$$

or an alternative iodate reduction reaction,

(7) $${\text{IO}}_{{}_{3}}^{-}+6{\text{H}}^{+}+6{\text{e}}^{-}\to {\text{I}}^{-}+3{\text{H}}_{2}\text{O}\;E{}^{\circ}=1.085\;\text{V}\text{.}$$

The chemical reactions of Cl_2_, Br_2_, and I_2_, when exposed as gases to Si surfaces, are known to preferentially attack step sites [26]. In semiconductor processing, this reaction is carried out at high temperatures; nonetheless, an analogous reaction in solutions would be expected to destroy por-Si. The high surface area and defect-laden surfaces of por-Si would be much more susceptible to such reactions in comparison to a polished silicon surface. Therefore, it is not surprising that the production of halogens in solution should lead to the destruction of por-Si. That Br_2_ destroys por-Si was previously reported by Kelly and co-workers [27].

Now, we turn to the etch chemistry of I^–^, I_2_, and I_3_^–^ with polished Si and por-Si surfaces. To do so, we made up a series of solutions from HI, I_2_, HI + I_2_, or NaI + I_2_. These were dissolved either in water or in water/ethanol solutions and exposed to mirror finish Si(100) and Si(111) surfaces. The Si surfaces were hydrogen terminated as their oxide layers were stripped by etching first in HF. Alternatively, a layer of por-Si was produced with either a FeCl_3_**·**6H_2_O + HF or a V_2_O_5_ + HF solution. The layer exhibited a uniform blue or green color indicative of a homogeneous por-Si film. Immediately after being made and rinsed, these layers were exposed to this set of solutions. No etching of either the polished Si surfaces or the por-Si films was noted. Iodide, iodine, and triiodine solutions do not react with either flat, defect-free surfaces or the high-surface-area, defect-laden surfaces of por-Si when HF is not also added to the solution. This result is consistent with the work of Haber et al. [25] who showed that I_2_/methanol solutions can lead to the formation of Si-I and Si-OCH_3_ bonds; however, they gave no indication for an increase in surface area or other signs of etching. That iodide-containing but HF-free solutions can affect the surface of silicon or por-Si but do not etch the surface is also consistent with other reports of solar cell characteristics and flatband potentials [28,29] or photoluminescence properties [30] that shift upon exposure to iodide- or triiodide-containing solutions.

**Figure 3:**
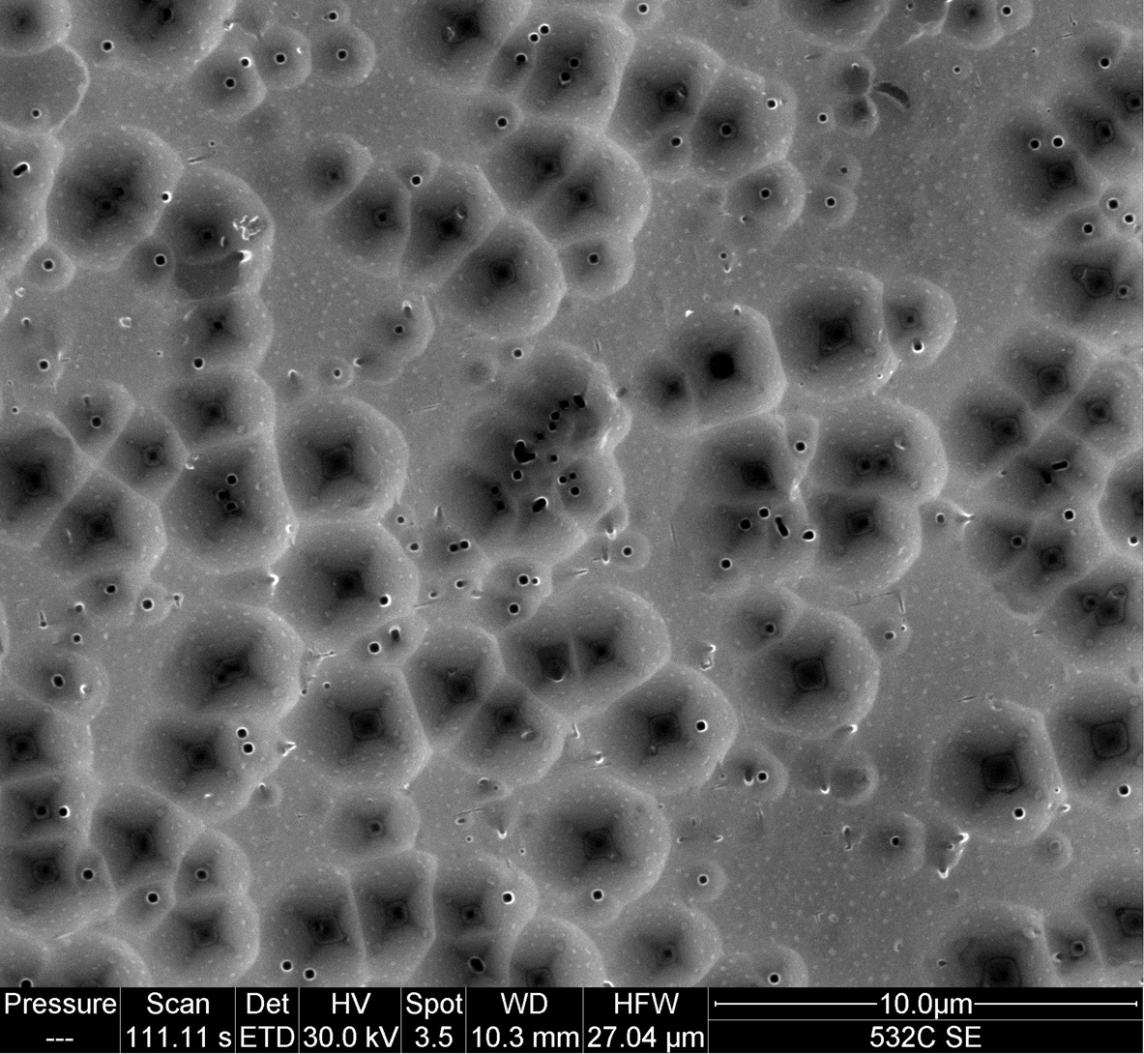
**SEM micrograph of 0- to 100-**Ω **cm p-type Si(100) etched in I**_**2**_ **+ NaI + ethanol + HF for 25 min.**

We investigated the etch behavior of solutions containing I^–^, I_2_, and I_3_^–^ to which HF has also been added. Neither HI + HF nor I_2_ + HF solutions exhibit appreciable reactivity with polished Si surfaces. We note no bubbling on the faces of the crystals, and the surfaces retain their mirror finishes after rinsing. Occasionally, a few small bubbles may appear after many minutes on scratches or the edges of the crystals. This indicates that there is some reactivity with defects but very low reactivity with well-ordered terraces. An I_3_^–^-containing solution can roughen a polished Si surface. This is a slow process requiring about half an hour or more. As shown in Figure 3, this etch process also exhibits a degree of crystallographic anisotropy. We exposed a p-type Si(100) crystal to, for example, a 0.005 M I_2_ + 0.04 M NaI in 3:1 ethanol/HF solution or an 0.004 M in I_2_, 0.02 M in NaI in 25% HF. Most of the etch pits are circular. However, as shown in Figure 3, some of the pits gradually convert to inverted pyramids, often with approximately 100-nm circular pits at their apex. The pyramids are 750 nm to 2 μm on a side. There is no evidence of nanoporous silicon on such samples. No bubbles formed during etching. This may be related to the slow etch rate.

Now, we address the etch chemistry of por-Si films with solutions containing I^–^, I_2_, and I_3_^–^ to which HF has also been added. HI + HF, I_2_ + HF + ethanol, and HI + I_2_ + HF solutions all remove por-Si layers accompanied by the formation of bubbles. The rate at which the por-Si is destroyed is dependent on the concentration of the iodine species. Interestingly, when I^–^ and I_2_ destroy por-Si, they leave behind a nearly mirror-like Si substrate. These two species, therefore, facilitate step flow etching in HF solutions. This is consistent with their lack of reactivity with the terraces of polished surfaces. I_3_^–^-containing solutions, on the other hand, remove por-Si to reveal a rough and pitted surface much like the one shown in Figure 3. Again, this is consistent with the more aggressive and anisotropic nature of I_3_^–^-induced etching. All three of these species destroy nanoscale Si structures; therefore, the initiation step and etch rate of each reaction must not be constrained by quantum confinement.

These results are general for the other halogens and halogenates. A 0.04-M Br_2_ + HF solution led to etching of flat Si surfaces, which remained flat after etching. If the solution was exposed to a por-Si layer, the por-Si was removed. As mentioned above, this is consistent with the work of Bressers et al. [27]. A solution of 0.07 M NaBrO_3_ + HF also etched Si. By the solution color change observed, it was clear that the reaction of BrO_3_^–^ generated Br_2_ in solution. As expected, the etching of a polished Si surface led to a flat surface after etching. Similarly, a 0.2-M KClO_3_ + HF solution led to the etching of polished silicon, which resulted in the formation of Cl_2_ in solution and a flat final surface. Neither one of these oxidants etched Si with the same degree of anisotropy as observed with iodate. This may be due to the more reactive nature of Br_2_ and Cl_2_ as compared to I_2_ and the lack of a species analogous to I_3_^–^.

## Conclusions

Etching of silicon in iodate solutions is extremely complex and difficult to control. Surface structures including pits (circular, triangular, or rectangular), pyramids (erect or inverted), roughness, and pits within pits or pits between pyramids have been obtained. This complexity is engendered by a competition of several isotropic and anisotropic chemical and electrochemical reactions of IO_3_^–^, I_3_^–^, I_2_, and I^–^. Whereas I_2_ and I^–^ much prefer to attack defect and step sites leading to flat surfaces, I_3_^–^ is able to attack terrace sites and exhibits a degree of crystallographic anisotropy as evidenced by the production of crystallographically defined features. We believe that the extremely high etch rates induced in IO_3_^–^ solutions are caused by IO_3_^–^ being able to increase the surface area and defect density of the Si substrate through the formation of porous silicon. Whereas a substantial porous layer might be formed in a non-recirculating flow through etch system, porous Si is subsequently destroyed by the actions of I_3_^–^, I_2_, and I^–^ as these reaction by-products build up in the etchant. Bromate and chlorate etching are not as complex. However, these only lead to flat surfaces at least in part due to the higher reactivity of Br_2_ and Cl_2_ in solution, both of which lead to step flow etching.

## Competing interests

The authors declare that they have no competing interests.

## Authors’ contributions

Both authors designed, analyzed, and performed the experiments. KWK was primarily responsible for the writing of this report. Both authors read and approved the final manuscript.
